# RNA modifications in insects

**DOI:** 10.3389/finsc.2024.1448766

**Published:** 2024-08-26

**Authors:** Yaoyu Jiao, Subba Reddy Palli

**Affiliations:** ^1^ Department of Entomology, Martin-Gatton College of Agriculture, Food and Environment, University of Kentucky, Lexington, KY, United States; ^2^ Department of Genetics, Yale School of Medicine, New Haven, CT, United States

**Keywords:** m^6^A, epigenetics, insect development, environmental adaptation, reproduction

## Abstract

More than 100 RNA chemical modifications to cellular RNA have been identified. *N* ^6^-methyladenosine (m^6^A) is the most prevalent modification of mRNA. RNA modifications have recently attracted significant attention due to their critical role in regulating mRNA processing and metabolism. tRNA and rRNA rank among the most heavily modified RNAs, and their modifications are essential for maintaining their structure and function. With our advanced understanding of RNA modifications, increasing evidence suggests RNA modifications are important in regulating various aspects of insect life. In this review, we will summarize recent studies investigating the impact of RNA modifications in insects, particularly highlighting the role of m^6^A in insect development, reproduction, and adaptation to the environment.

## 1 Introduction

RNA plays pivotal roles in various cellular processes, including transcription and translation. Unlike DNA, which always exists in a double-stranded structure, RNA molecules are typically single-stranded and self-fold into the functional conformation with a three‐dimensional structure (e.g., transfer RNA, tRNA, and ribosomal RNA, rRNA). Furthermore, RNA molecules are subject to significantly more chemical modifications of their bases compared to DNA, with over 100 RNA modifications identified to date (1). Among major classes of RNA, transfer RNA (tRNA) contains the highest proportion of RNA modifications, with approximately 17% of all tRNA nucleotides being modified (2). Ribosome RNA (rRNA) comes in second, with around 2% of nucleotides being modified (3). Many rRNA modifications are located at the functional sites and are critical for their function (3–5). Additionally, many tRNA and rRNA modifications exhibit conservation across different organisms (2, 6). Historically, the function of mRNA modifications was poorly understood. The rapid development in genome-scale detection methods enables the accurate mapping of several mRNA modifications such as *N* ^6^-methyladenosine (m^6^A), 5-methylcytidine (m^5^C), pseudouridine (Ψ), and N4-acetylcytidine (ac^4^C), greatly enhancing our understanding of mRNA modifications (7–12). Among them, the m^6^A modification is the most prevalent mRNA modification and the most intensively studied so far, with many important functions of m^6^A in cellular and biological processes having been discovered (13–20).

Writers, readers and erasers are the three most important groups of proteins regulating the dynamics and function of RNA modifications. Writers are responsible for installing the RNA modifications. Some writers are functional when they form a complex with other writer proteins or RNAs. For example, rRNA 2’-*O*-methylation and pseudouridine (Ψ), the two most abundant modifications in rRNA, are installed by small ribonucleoproteins (sRNP) complexes. The mRNA m^6^A transmethylase complex, the best understood mRNA modification writer, comprises the heterodimeric enzymes Mettl3 (Methyltransferase 3) and Mettl14 (Methyltransferase 14), as well as other accessory proteins, including WTAP (Wilms tumor 1-associated protein), VIRMA (Vir Like M6A Methyltransferase Associated), RBM15 (RNA-binding motif protein 15), ZC3H13 (zinc finger CCCH-type containing 13), and Hakai (21–23). RNA modification writers can also be stand-alone enzymes, including those that are responsible for installing many rRNA modifications such as *N* ^1^-methyladenosine (m^1^A), m^5^C, *N* ^7^-methylguanosine (m^7^G), 3-methyluridine (m^3^U) and a few mRNA modifications such as m^5^C, Ψ, and ac^4^C (3, 24). Reader proteins can directly recognize the RNA modifications or indirectly bind to the RNAs that undergo structure change after modifications, execute the biological function and regulate RNA metabolisms. The study of RNA modification readers mainly focuses on mRNA modifications. Among mRNA modification readers, the function and mechanisms of m^6^A mRNA readers are the most well understood. The mRNA m^6^A readers are mainly YTH family proteins, distributed in both cell cytosol and nucleus (25). Readers for other mRNA modifications such as m^1^A, m^5^C and ac^4^C have also been reported, but their binding mechanisms and regulatory roles are not yet clear (24). Lastly, eraser proteins can remove RNA modifications and reverse them back to an unmodified state. The existence of erasers has important implications, as it suggests that RNA modifications can serve as an epigenetic pathway to regulate gene expression bidirectionally. Compared to tRNA and rRNA, the mRNA is more dynamic and serves as the mediator of gene expression. Thus, the regulation of mRNA through modifications is more straightforward and has greater potential for understanding development and disease. Like readers, mRNA erasers are the most studied and understood in the mRNA m^6^A pathway. Two m^6^A demethylases, FTO (fat mass and obesity-associated) and ALKBH5 (alkB homolog 5, RNA demethylase), have been identified in mammals (17, 22). One potential m^6^A eraser, ALKBH8, was recently identified in insects (26).

RNA modifications play critical roles in biological functions. Previous studies in *Escherichia coli* bacteria and *Saccharomyces cerevisiae* yeast have suggested that tRNA and rRNA modifications are important in the formation and maintenance of their structure, with some modifications occurring at the key nucleotide within functional active sites (2–4). In the past decade, our understanding of mRNA modifications, particularly m^6^A, has experienced explosive advancements. The m^6^A regulates nearly every step of mRNA maturation and activity, including splicing (27, 28), polyadenylation (29), nuclear export (30, 31), stabilization (32), degradation (33) and translation (34). Therefore, m^6^A plays a critical role in regulating gene expression and affecting animal physiological processes by directly acting on mRNAs. Several reviews have exquisitely addressed the molecular mechanisms and therapeutic potentials of m^6^A (22, 25, 35).

Most of our understanding of RNA modifications including tRNA, rRNA and mRNA modifications comes from model organisms such as *E. coli*, *S. cerevisiae*, mice, zebrafish, and fruit flies (2, 4, 10, 36, 37). It remains largely unexplored about the specific role and mechanism of RNA modifications in the development and physiology of non-*Drosophila* insects. As the most successful animal group, insects develop in diverse and distinguished patterns, have astonishing behaviors, and adapt to almost every corner of the earth (38). It is anticipated that RNA modifications, which are crucial for regulating cellular function including gene expression, play critical roles in different aspects of insect biology. In the last few years, entomologists have begun to investigate the role of mRNA m^6^A in different insects, uncovering various functions of m^6^A in insects. This review aims to present an overview of recent advances in understanding RNA modifications in insects, specifically focusing on mRNA m^6^A which has been suggested to regulate insect development and the response of insects to the environment. The final section of this review also addresses the functions of tRNA and rRNA modifications in insects. We did not cover other non-m^6^A mRNA modifications and other RNA modifications due to the limited number of studies on these modifications in insects.

## 2 The role of m^6^A RNA modifications in insect development and reproduction

Insects exhibit diverse life cycles, but most holometabolous insects adhere to a general pattern, including egg, larva, pupa, and adult life stages. The cycle begins with the eggs laid by adults in a desirable environment. After completing embryonic development, larvae hatch from the eggs and undergo a rapid growth phase with massive feeding and several molts as they significantly increase body size. Following the larval stage, insects undergo pupation with extensive tissue remodeling and transform into pupae. Finally, the adults emerge from pupal cases, complete their reproductive development, and are ready to produce the next generation. The hemimetabolous insects skip the pupal stage as the nymphs undergo metamorphosis to become adults. Insect development and reproduction require the temporal and spatial regulation of gene expression to meet the needs of different tissues at various stages. As a critical epigenetic factor, the role of m^6^A in mRNA metabolism during insect development and reproduction is an intriguing question.

### 2.1 Embryogenesis

During the first few hours of embryogenesis, the maternal RNAs play a critical role in embryonic development. As the development progresses, the zygotic genome starts to synthesize its own RNAs. In vertebrate embryonic development, the m^6^A pathway is important by regulating both maternal and zygotic mRNAs. Notably, a significant portion of maternal mRNAs in the embryos of zebrafish and mice are marked with m^6^A labels, which are critical for the maternal-to-zygotic transition (MZT) (37, 39). Several studies also have highlighted the importance of m^6^A in post-MZT embryonic development (16, 40). Disturbing zygotic m^6^A by knocking out the *Mettl3* gene in the embryo caused death at an early embryonic stage in mice by failing to regulate naïve pluripotency (16). Similarly, morpholino-mediated knockdown of *Mettl3* in zebrafish embryos caused tissue differentiation defects and increased apoptosis (40).

In insects, m^6^A modifications on maternal mRNA are also important for successful embryonic development. In *Drosophila melanogaster*, m^6^A has been shown to contribute to the degradation of maternal RNAs (41). A study utilizing RNA-seq analysis of embryos derived from both wild type and *Mettl3–Mettl14* double maternal mutants revealed that, in the eggs laid by the mutants, a significant portion of m^6^A-marked maternal RNAs remains stable during the maternal-to-zygotic transition (41). Approximately 40% of eggs laid by *Mettl3-Mettl14* double maternal mutants failed to hatch (41). A more severe effect was observed in the red flour beetle *Tribolium castaneum;* eggs laid by *Mettl3* dsRNA-treated females stopped embryonic development at an early embryonic stage before nuclear division and migration (42).

The role of m^6^A in post-MZT embryonic development varies in different insects. *Mettl3* null mutants of *D. melanogaster* remain viable and can survive to adult stages (27). However, in the silkworm *Bombyx mori*, knockdown of *Mettl3* by embryonic injection of siRNA affected several signaling pathways, including Wnt and Toll/Imd signaling required for embryogenesis (43). Similarly, in the fall armyworm, *Spodoptera frugiperda*, methylated RNA immunoprecipitation-seq, and RNA-seq suggested that m^6^A regulates the expression of genes required for organogenesis during embryogenesis (44).

### 2.2 Post-embryonic development

After hatching from eggs, insect larvae undergo several molts and metamorphosis and ultimately develop into the adult stage with reproduction capability. Insects exhibit a remarkable diversity in their post-embryonic development modes, including different molting times, various body structures, specialized organs, and unique behavioral characteristics. These distinct features are shaped by determinantal genes, which are precisely expressed and regulated to ensure accurate and appropriate development. As a major epigenetic marker, mRNA m^6^A regulates insect post-embryonic development. While m^6^A deficiency does not cause acute lethal effects in the post-embryonic stage of insects (17, 42), recent research has uncovered its role in fine-tuning gene expression and delicately moderating post-embryonic development in various insect species.

Several recent studies suggest that m^6^A plays a critical role in caste differentiation in social insects. The western honeybee, *Apis mellifera*, is a widely used model for studying the development and behaviors of social insects. A recent investigation has found a critical role of m^6^A modification in larval development and caste differentiation of honeybees (45). For the genetically identical female larvae of honeybees, worker larvae contain hypermethylated m^6^A than queen larvae (45). Chemical suppression of m^6^A in worker larvae can induce the development of queen features (45). In addition, another group showed that the m^6^A pathway might regulate the behavior of honeybee workers; different *Mettl3* expression and m^6^A levels have been observed in the brain and fat body of young and old adult honeybee workers inferring the role of m^6^A in regulating behavior switch during age progression (46, 47).

**Figure 1:**
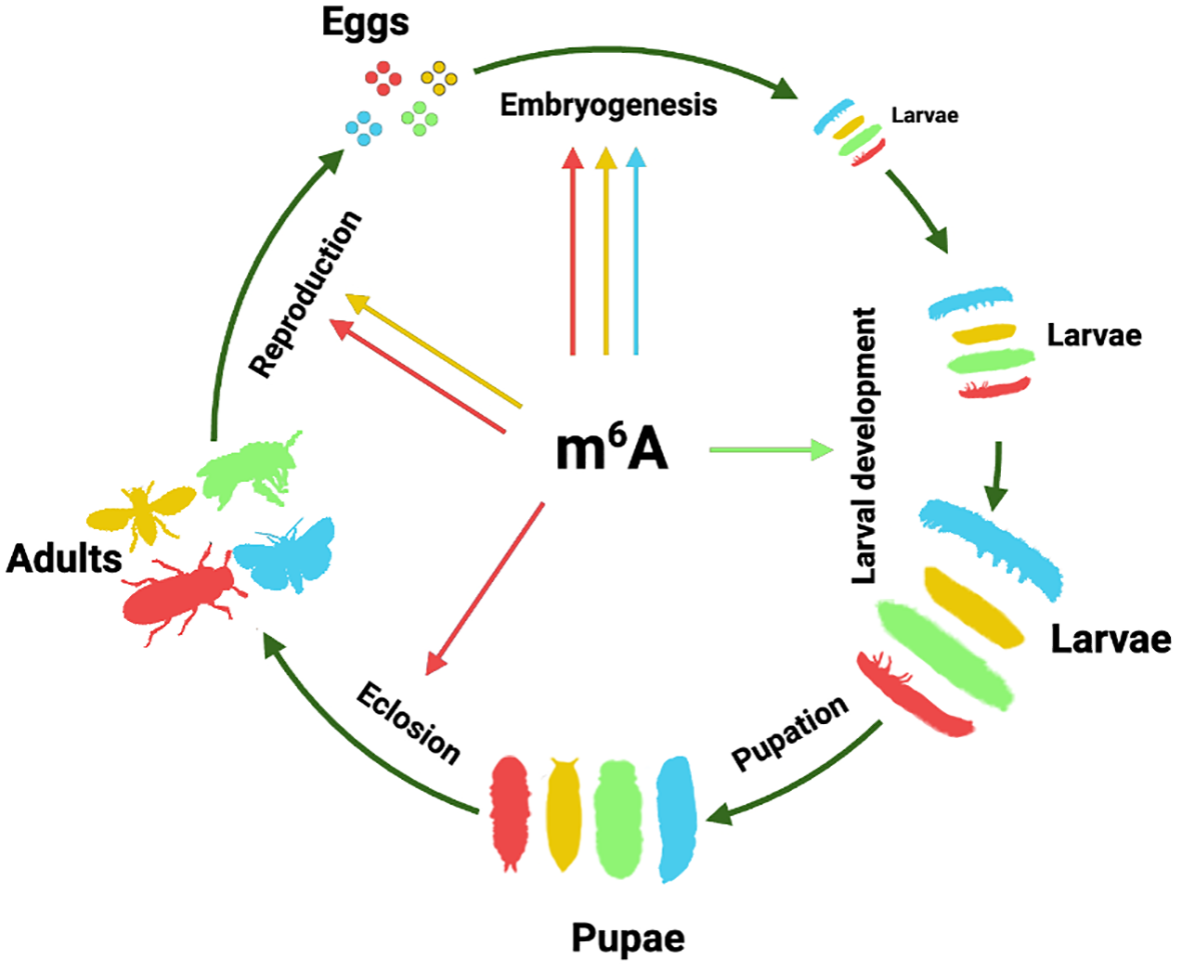
The role of m^6^A RNA modification in insect development and reproduction. Hypothetical insect life cycles are used to represent the function of m^6^A in various developmental stages of different insects. Yellow represents Diptera drawing with the silhouette of *D. melanogaster*; green represents Hymenoptera using *A. mellifera* silhouette; red represents Coleoptera using *T. castaneum* silhouette; and blue represents Lepidoptera using *S. frugiperda* silhouette. The arrow sourced from the central m^6^A symbol indicates the existence of reported evidence in this insect order. The silhouettes were obtained from PhyloPic (http://phylopic.org) or created by the authors.

As mentioned above, *D. melanogaster* is the most extensively studied insect regarding the function of m^6^A (17, 21, 27, 36, 48, 49). Deficiency of *Mettl3* does not cause lethal effects in *D. melanogaster* post-embryonic development (17). However, *Mettl3* mutant flies show defects in locomotion and memory, which are mediated by different m^6^A readers (17, 48). These behavior and cognitive defects are consistent with the detected high enrichment of m^6^A in the *D. melanogaster* nervous system (49). The effect of m^6^A on fly behavior may be induced by disturbed axonal overgrowth and misguidance of the fly nervous system (50). More interestingly, *D. melanogaster* sex determination is also regulated by the m^6^A pathway (17, 27, 49). The splicing of *Sex-lethal (Sxl*, the master sex determination factor) mRNA is regulated by the m^6^A pathway. Females with m^6^A deficiency produce male-specific Sxl isoform and have relatively high mortality, while males are unaffected (17, 49). The regulation of sex determination seems to be specific for *D. melanogaster.* The function of m^6^A in regulating sex determination was not observed in insects such as *T. castaneum* that do not utilize Sxl as a sex determination regulator (42).

m^6^A also affects various aspects of other insects. In *T. castaneum*, the m^6^A pathway plays a crucial role in the ecdysis process during eclosion; knocking down m^6^A writers causes the failure of shedding old pupal cuticles without affecting the formation of adult structures (42). In the silkworm *B. mori*, m^6^A was found to specifically mediate the function of the posterior silk gland regulated by juvenile hormone analog (51). In summary, the role of m^6^A in insect post-embryonic development appears to be very diverse and species-specific (**Figure 1**).

### 2.3 Reproduction

The m^6^A-mediated epigenetic regulation has been reported to be involved in many reproductive diseases of mammals (52). In mice, disturbing the m^6^A pathway by knocking out m^6^A players has been shown to affect the fertility of both males and females (29, 39, 53–55). The development of insect reproductive systems also requires the m^6^A pathway. In *D. melanogaster*, ovary development defects were observed in *Mettl3* mutants (17, 56). Meanwhile, male adults of *Drosophila Mettl3* mutants showed reduced fertility, and the effect of m^6^A on sperm spermatogenesis is likely mediated by regulating *chickadee* mRNA (57). Similarly, in *T. castaneum*, the knockdown of *Mettl3* affects the fertility of both females and males; the reduced number and size of female oocytes were observed in *Mettl3* knockdown females (42). A recent transcriptome-wide study conducted in *Anopheles sinensis* revealed that several genes associated with spermatogenesis are m^6^A-modulated and may be regulated by the m^6^A pathway (58). While existing studies indicate a broad involvement of m^6^A in regulating insect reproduction, the precise mechanisms through which m^6^A influences the development of insect reproductive systems and whether it acts through shared pathways or genes in various insect species remain unclear.

## 3 The role of m^6^A RNA modifications in insect stress response

**Figure 2:**
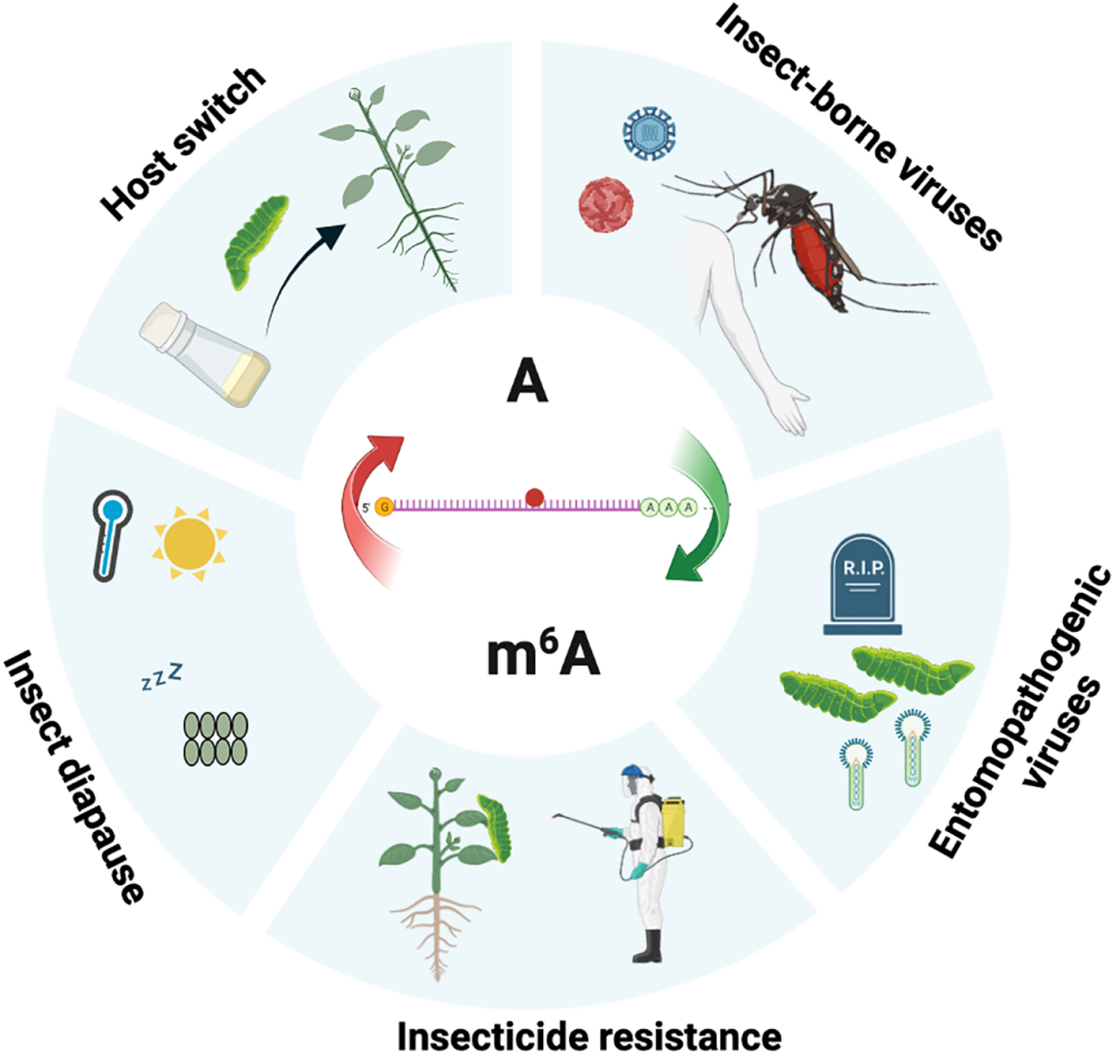
The role of m^6^A modification in insects interacting with external factors. Schematic representation shows the involvement of m^6^A in the insect adaptation to the environment. This figure was created utilizing Biorender.

Insects are confronted with a wide range of external factors, including both biotic and abiotic factors, and have evolved various strategies to survive and thrive under these challenging conditions (59–62). Recent studies have shed light on the involvement of the m^6^A pathway in the adaptive responses of insects to various external challenges, including virus infections and exposure to insecticide (**Figure 2**).

### 3.1 Entomopathogenic viruses

Entomopathogenic viruses are one of the major threats to insect colony maintenance and farming, but meanwhile, serve as a promising pest control strategy. The silkworm, *B. mori*, is an economically and culturally important insect in Asia. *B. mori* nucleopolyhedrovirus (BmNPV) is one of the top threats to the silkworm industry. The m^6^A immunoprecipitation sequencing revealed that m^6^A levels of *B. mori* are associated with BmNPV infection (63, 64). A significant increase in m^6^A peaks was observed in *B. mori* midguts following BmNPV infection (63, 64). However, contrasting functions of the m^6^A pathway in the BmNPV infection were observed. One investigation showed that knocking down *BmMettl3* in BmN cells led to an increased BmNPV infection, while overexpression reduced the infection (64). On the contrary, in another study (63), it has been shown that low expression levels of *BmMettl3* enhance the resistance to BmNPV infection. Despite the established involvement of m^6^A in the BmNPV infection of silkworms, the precise mechanisms through which m^6^A mediates the entomopathogenic virus infection remain elusive.

### 3.2 Insect-borne viruses

Unlike entomopathogenic viruses, insect-borne viruses typically do not significantly affect insect survival; however, they pose a significant threat to food safety and human health by causing plant and human diseases (65, 66). Recent studies have shed light on the regulatory role of the m^6^A pathway in viral proliferation by acting on viral RNA or the host’s genes (67–70).

The presence of m^6^A sites has been identified in the RNA genomes of *Flaviviridae* viruses, including dengue, Zika, yellow fever, and West Nile virus (71). It has been revealed that the m^6^A machinery of the human host can directly regulate the viral replication by modulating RNA modifications of the Zika viral mRNA; the methylation of viral mRNA promotes the degradation of viral mRNA mediated by YTHDFs (69). A later study has further shown that *Flaviviridae* infection also induces the m^6^A changes in human cellular mRNAs, which regulates the viral infection (68). Similarly, within the mosquito host, the m^6^A pathway of mosquitos also plays a regulatory role in virus proliferation (67). In the yellow fever mosquito, *Aedes aegypti*, the m^6^A pathway in mosquitoes has been found to regulate the proliferation of dengue virus (67). The regulation of m^6^A modification is mediated by affecting *ubiquitin carrier protein 9* (*Ubc9*) gene expression of *Ae. aegypti*; knockdown and overexpression of *Ubc9* led to reduced and increased dengue virus infection *(* 67 *)*. Inhibition of m^6^A, either by applying a chemical inhibitor of m^6^A or silencing m^6^A methyltransferase genes, reduced the abundance and stability of *Ubc9* mRNA and thus decreased dengue virus infection (67). A case of m^6^A-mediated regulation of plant pathogenic virus replication in insect vectors has also been reported (72). The study showed that the infection of rice black-streaked dwarf virus (RBSDV) decreases the m^6^A level in midgut cells of the insect vector, small brown planthopper, *Laodelphax striatellus*. Silencing of m^6^A methyltransferase in *L. striatellus* significantly induced RBSDV titer; however, it is not clear how m^6^A mediates the replication of RBSDV (72).

In recent reports, the role of m^6^A in insect vector-virus interactions has been highlighted. However, conflicting roles of m^6^A in the virus life cycle have been observed. This discrepancy may arise from different modes of action, such as directly acting on viral RNA or through targeting genes of the insect host that are critical for virus infection. Since many insect-borne viruses are RNA viruses, they are likely subject to modifications by the m^6^A pathway of insect hosts and may further be regulated by the m^6^A pathway. We anticipate that further investigations will uncover additional roles of the host and vector m^6^A pathways in regulating insect-viruses interactions.

### 3.3 Other external factors

Besides viruses, recent studies have also revealed the involvement of the m^6^A pathway in insects’ response to other external stresses. Many insects can rapidly develop pesticide resistance through natural selection, causing tremendous economic and environmental losses (73). A recent study indicated that the m^6^A pathway contributes to the insecticide thiamethoxam resistance in a global invasive agricultural pest, the whitefly *Bemisia tabaci* (74). m^6^A levels were found to be higher in resistance strains than the susceptible strain, and the introduction of a m^6^A site at a P450 gene promoter region by a mutation in the resistance strains is one of the main causes for the resistance emergence (74). The m^6^A pathway has also been revealed to be involved in the host plant adaption of diamondback moth *Plutella xylostella* (75). Compared to the wild-type insects, *P. xylostella* mutants of *Mettl3* and *Mettl14* showed a significant deduction of larval and pupal weight when transferring from the artificial diet to the host plant (75). m^6^A was also found to regulate juvenile hormone (JH) titer in the diamondback moth *P. xylostella* to enhance the pest fitness when resisting the Bt Cry1Ac toxin (76).

Insect diapause is a crucial and adaptive trait that enables insects to survive in extreme environments, usually triggered by environmental factors such as photoperiod and low temperature (77). A study on silkworm *B. mori* showed that the diapause‐destined eggs have a relatively greater m^6^A content than nondiapause‐destined eggs (78). The follow-up studies from the same group further revealed that the m^6^A pathway regulates the diapause of *B. mori* eggs by acting on genes in the insect hormones synthesis pathway (79, 80).

## 4 tRNA and rRNA modifications in insects

Despite the important roles that mRNA modifications play, among all RNA types, tRNA has the highest proportion of modifications, followed by rRNA. Many modifications of tRNA and rRNA are in the functional regions and thus are critical for maintaining RNA structure and facilitating their functions (2–5, 81). The loss of these important modifications can result in translation deficiency and cause developmental delay and/or lethal effects. Some modifications are installed by non-conserved enzymes or altered in response to cellular environmental change, playing less critical roles in translation (3, 81). However, the functions of these modifications in insects including the model insect *D. melanogaster* are not well studied. Given that tRNA and rRNA have the most abundant modifications, understanding how these modifications affect insects could provide valuable insights into insect development and developing pest control strategies. Therefore, in this section, we summarize the studies that have investigated various tRNA and rRNA modifications in insects, although there are a limited number of them.

### 4.1 Transfer RNA modifications

Studies in *D. melanogaster* have focused on specific tRNA modifications and shown that some are critical for *Drosophila* development and maintenance of normal lifespan. For example, *N* ^6^-threonylcarbamoyl-adenosine (t^6^A) is a universally conserved modification in the tRNA anticodon region, which is critical for decoding ANN codons and supports proper translation (82). Loss of t^6^A induced by knockdown or mutations of t^6^A biosynthesis factors caused various developmental deficiencies, including melanotic growth and reduced developmental rate (83, 84). At the tissue level, proliferating tissues, such as wing discs, are more affected by t^6^A deficiency than non-proliferating tissues (83, 84). In a recent study, research through a genome-wide screen revealed that two tRNA 2’-*O*-methylation methyltransferases are critical for small RNA pathways (85). Mutants are viable and fertile but exhibit a shorter lifespan and increased sensibility to RNA virus infection (85).

The tRNA modifications are involved in mosquito reproduction (86). The abundance of tRNA modifications is sex-associated in mosquitoes (86). Higher tRNA modifications were detected in females compared to males of *Ae. aegypti*, *Culex pipiens*, and *Anopheles stephensi* mosquitoes (86). The high tRNA modifications in females likely are contributed by the increased expression of tRNA-modifying enzymes in female ovaries, suggesting tRNA modification may be critical for mosquito reproduction (86).

### 4.2 Ribosomal RNA modifications

2’-*O*-methylation and pseudouridine (Ψ) are the two most abundant rRNA modifications, which are modified by box C/D and box H/ACA sRNP complexes, respectively. Many of these modifications are clustered in the functional region of the ribosome and thus are critical for cell function and survival (87, 88). *D. melanogaster* null mutants of the box H/ACA Ψ synthetase *Nop60B* results in lethality, while partial loss-of-function mutations of *Nop60B* caused developmental delay, reduced body size, hatched abdominal cuticle, and decreased female fertility (89, 90). In *T. castaneum*, knockdown of box C/D methyltransferase *fibrillarin* (TC002569) and the box H/ACA Ψ synthetase *TcNop60B* (TC010569) causes high mortality (unpublished data from the authors) (91).

Besides 2’-*O*-methylation and Ψ, rRNA contains many other modifications, including methylation, acetylation, etc. These rRNA modifications are usually introduced by stand-alone enzymes (3). For example, in *D. melanogaster*, methyltransferase NSUN5 was predicted to modify cytosine 3620 of 28S rRNA (92). Loss of NSUN5 can increase the lifespan and stress resistance of *D. melanogaster* (92). This is likely due to the loss of methylation, causing the ribosome structure to change and making ribosomes favor the recruitment of oxidative stress-responsive mRNAs (92). *D. melanogaster* Mettl5 is specifically required to deposit m^6^A near the decoding center of 18S ribosomal RNA (93). However, Mettl5 does not affect ribosome maturation but is required for the orientation and locomotion of adult flies (93).

## 5 Conclusions and future perspectives

In the past few years, RNA modifications, including mRNA m^6^A and other RNA modifications, have been revealed to be fundamental in RNA transcription and translation, which add another layer of epigenetic factors for gene regulation (1). Among RNA modifications, the function of mRNA m^6^A in insects is most well-understood and plays an important role in the regulation of the insect life cycle. Loss of function studies such as knockdown or knockout of the key players in the m^6^A methyltransferases complex showed that the m^6^A pathway is critical for insect embryogenesis and reproduction, and the regulation is likely conserved among insects. However, the impact of the m^6^A pathway on insect post-embryonic development is either a sublethal/minor effect or insect species dependent. Furthermore, more and more studies suggest that m^6^A is likely involved in insects’ environmental adaptation when insects face biotic or abiotic environmental factors such as virus infection and natural and artificial xenobiotics, which suggests that the mRNA m^6^A could serve as a critical factor in understanding the mechanisms behind insect pest outbreaks and evolution. In future studies, it is important to understand how m^6^A regulates different insect life processes. The genes affected by m^6^A could serve as new targets for pest control or pesticide resistance management.

Research on the function of tRNA and rRNA modifications in insects is generally limited and has been mostly focused on *Drosophila*. However, many of these modifications serve as important functional sites in tRNA and rRNA, and their deficiency often causes severe lethal effects. Thus, genes involved in tRNA and rRNA modifications could serve as potential targets for RNAi pest control strategies.
